# The Effects of Gender and Family Wealth on Sexual Abuse of Adolescents

**DOI:** 10.3390/ijerph16101788

**Published:** 2019-05-20

**Authors:** Eyglo Runarsdottir, Edward Smith, Arsaell Arnarsson

**Affiliations:** 1 School of Education, University of Iceland, 105 Reykjavík, Iceland; er@hi.is; 2 Prevention Research Center, Penn State University, State College, PA 16801, USA; eas8@psu.edu

**Keywords:** gender, family wealth, sexual abuse, adolescence

## Abstract

*Background:* Sexual abuse and sexual assaults against adolescents are among the most significant threats to their health and well-being. Some studies have found poverty to be a risk factor for sexual abuse. The present study investigates the effects of gender and family affluence on the prevalence of sexual abuse of 15-year-old Icelanders in the 10th grade. *Methods:* The study is based on data collected for the Icelandic part of the Health Behaviour in School-aged Children study in 2014. Standardized questionnaires were sent to all students in the 10th grade in Iceland, of which 3618 participated (85% of all registered students in this grade). *Results:* Girls were more than twice as likely to be sexually abused as boys (20.2% versus 9.1%). Adolescents perceiving their families to be less well off than others were twice as likely to report sexual abuse as those of ample or medium family affluence. However, family affluence had more effect on the prevalence of abuse in girls than in boys. *Conclusion:* Female gender and low socioeconomic status may independently contribute to the risk of sexual abuse.

## 1. Introduction

Sexual abuse and sexual assaults against adolescents are among the most significant threats to their health [1]. The World Health Organization (WHO) has defined sexual abuse during childhood and adolescence (CSA) as “the involvement of a child in sexual activity that he or she does not fully comprehend, is unable to give informed consent to, or for which the child is not developmentally prepared and cannot give consent, or that violates the laws or social taboos of society. Child sexual abuse is evidenced by this activity between a child and an adult or another child who by age or development is in a relationship of responsibility, trust, or power, the activity being intended to gratify or satisfy the needs of the other person” ([2], page 75). Researchers differ in their definitions of sexual abuse. Variables such as age of the abused, age of the abuser, the nature of the involvement, and timeframe can partly explain the differences in prevalence from different studies [3,4]. Prevalence also seems to differ by geographical context and cultural background, showing lower rates of reported sexual abuse amongst boys and girls in Asia and South America and higher rates in Australia and New Zealand [3].

Different studies have reported a varied prevalence of sexual abuse in adolescents. A study of 10th graders in Iceland shows that 15% of them had experienced some form of abuse, and, of these, two thirds had suffered abuse more than once [5]. A study in an older sample of 16- to 19-year-olds in Iceland had previously shown 36% of girls and 18% of boys reporting CSA [6]. These results are in line with a Swiss study [7] stating that 40% of girls and 17% of boys reported CSA and a U.S. meta-analysis showing that 30% of girls and 15% of boys reported CSA [8]. However, these findings are significantly lower than those in a Swedish study [9], where 65% of girls and 23% of boys reported CSA. A global meta-analysis estimates the prevalence of self-reported CSA for girls at between 16% and 20% (lower and upper limit) and for boys, between 7% and 9% [3]. 

Variations in approach could partly explain the difference in prevalence of CSA because studies with self-reported sexual abuse usually show higher prevalence, especially for girls, than studies based on official statistics [3]. In addition, studies amongst older adolescents also state higher prevalence because of higher rates of dating violence and peer assault [5,6]. All the studies mentioned above report girls experiencing sexual abuse at higher rates than boys, with girls experiencing three to five times the rate of sexual abuse, compared to boys [10,11,12]. 

Numerous studies show the impact of poverty or low socioeconomic status on the development and well-being of adolescents [13,14,15,16,17]. A recent report from the Health Behaviour in School-aged Children (HBSC) study [18] found that differences in family affluence continue to have a strong effect on adolescent health and well-being. These findings showed that adolescents from low-affluence families had poorer health, lower life satisfaction, higher levels of obesity and sedentary behaviors, weaker communication with their parents, less social interaction via social media, and lower levels of support from friends and family. Many of these inequalities will have persistent life-long effects. Research results suggest that these inequalities may be increasing, and that the gap in several key areas of adolescent health is widening [19].

Some other studies have indicated that family affluence may have different effects on the outcomes of adolescent boys, compared to girls. For example, in Norway, Lien et al. [20] found socioeconomic status associated only with overweight and obesity in boys. Similar results have also been reported from a Swedish sample [21]. A Danish study showed that household income had more influence on the stress level in boys than in girls, while parental education was found to affect girls more than boys [22]. Several studies have found the association between low family socioeconomic status (SES) and mental problems in adolescence to be moderated by gender. Some studies claim that the relationship between low SES and high psychological distress is more evident among girls than among boys [23], while others report that boys are more sensitive to the effects of poverty than girls [24]. In a Finnish sample, Fröjd et al. [25] found that perceived financial difficulties were associated with depression for both girls and boys, but perceived financial difficulties were more prevalent among adolescent girls than boys. Other studies find no gender difference in the effects of SES on mental health [26].

When it comes to sexual abuse in adolescents, few studies have focused on the relationship between economic status (poverty or affluence) and CSA and the results have been inconsistent. Some studies have found poverty to be a risk factor for sexual abuse. Sedlak et al. [11] report that children from families with low SES were twice as likely to experience sexual abuse and three times as likely to be endangered than children from families with higher SES. In their recent study, Lee et al. [10] report a higher risk of severe and multiple types of abuse including sexual abuse for children experiencing poverty during childhood. This also affects overall health in adult years, especially for women. However, Oshima et al. [15] found no significant difference in CSA rates between more affluent and poor families, but a significant difference was reported between poor victims and wealthier victims of childhood sexual abuse for repeated reports of maltreatment to child protective services. 

Social and environmental explanations focus on how individuals are organized in society. Such explanations suggest that groups that are disadvantaged both economically and socially (e.g., females, the less affluent) suffer disproportionate exposure to stress [24]. The aim of this study was to investigate the effect of gender and family affluence on the prevalence of sexual abuse in Icelandic teenagers at the age of 15 (10th grade).

## 2. Methods

### 2.1. Participants

The study is based on self-reported data collected for the Icelandic part of the HBSC study in 2014. Standardized questionnaires were sent to all students in the 10th grade in Iceland, of which 3618 participated (85% of all registered students in this grade). 

### 2.2. Procedure

After a letter of introduction and a copy of the questionnaire had been sent to the principals of all elementary schools in Iceland, they were contacted and asked for permission. All except one gave permission, which meant that 171 schools participated. An information letter was then sent to all parents or custodians introducing the study and providing the opportunity to withdraw consent. Additionally, on the front page of the questionnaire, participants were informed about their right to refuse participation regardless of whether the schools and parents had given consent. The students filled out the questionnaire on paper in the classroom and returned their answers in unmarked envelopes that were collected by their teachers.

### 2.3. Materials

The students’ experiences of sexual abuse or assaults were assessed by asking them (a) “Has someone touched or fondled you in a sexual way when you did not want them to?”; (b) “Has someone made you touch their body in a sexual way when you did not want them to?“; (c) “Has someone attempted oral, anal, or vaginal intercourse with you when you did not want them to?”; or (d) “Has someone actually had oral, anal, or vaginal intercourse with you when you did not want them to?”. For all those four questions there were five possible answers: (1) “I refuse to answer”; (2) “Never”; (3) “Once”; (4) “A few times”; and (5) “Many times”. These items were included in the HBSC study from the Adverse Childhood Experiences questionnaire [27]. In this study, answers to these questions were grouped around two groups of students, i.e., those having experienced sexual abuse (in any form) or those that had not. 

Perceived family wealth was measured by asking “How well off do you think your family is?” The response categories were “Very well off”, “Quite well off”, “Average”, “Not so well off”, and “Not at all well off”. For this paper, these answers were lumped together to create three categories: “Well off”, “Average”, and “Not well off”. This three-category framework was designed as a proxy for young people’s perceptions of their family’s socioeconomic circumstances and implicates a subjective socioeconomic status. It is used frequently in similar studies and has previously been shown to reliably predict health inequalities [16,28,29,30]. It seems that subjective measures of socioeconomic status combine absolute and relative deprivation of current and past social circumstances along with future prospects [31,32].

### 2.4. Ethical Clearance Declaration

The data was collected anonymously but was reported to the Icelandic Data Protection Authority (No. S6463). This is a governmental institution that exercises surveillance over collection and processing of data in Iceland and ensures that data collected does not violate laws on data protection and privacy.

### 2.5. Data Analysis

Using IBM SPSS Statistics software (version 24.0, IBM, Armonk, NY, USA) we calculated descriptive statistics. Furthermore, logistic regression with odds ratio (OR) was used to explain relations between dependent and independent variables.

## 3. Results

Of the 3618 students (50.7% boys and 49.3% girls) responding to the question on sexual abuse, 14.6% (*N* = 528) said that they had experienced it (Table 1). A total of 3010 (85.4%) said that they had never been sexually abused, and 2.2% (80) specifically refused to answer these questions. We excluded the latter group from our analysis since our previous prevalence and risk study showed that the risk profile of these students was significantly worse than those that answered that they had never been abused [5]. Girls were more than twice as likely to be sexually abused as boys (20.2% versus 9.1%).

Table 2 shows that Icelandic adolescents who perceived their families to be less well off than others were twice as likely to report sexual abuse as were those of ample or medium family affluence. However, dividing these results by gender demonstrates that family affluence has more effect on the prevalence of abuse in girls than in boys. Among girls, 37.6% in the not well-off families had experienced sexual abuse, compared to 17.0% in the most affluent families.

We also looked separately at the four questions on sexual abuse, i.e., how often they had been against their will (a) touched in a sexual way; (b) made to touch someone else in a sexual way; (c) subjected to attempted rape; or (d) subjected to rape. We found a similar trend by gender and family wealth for each question (data not shown). 

Binary logistic regression was performed to assess the impact of gender and family affluence on the prevalence of sexual abuse. The results are shown in Table 3 with intercepts (B), standard error (SE), odds ratio (OR), confidence intervals (CI) and statistical significance. The first model containing the predictor variable of family affluence was statistically significant (odds ratio 1.24). Adding gender to the second model did not change the effect of family affluence but showed that gender also had a significant effect. The odds ratio of 2.4 indicates that girls are more than twice as likely as boys to be sexually abused. No significant interaction effects were detected between the two independent variables (chi-square 25.98; *p* = 0.96).

## 4. Discussion

In the current study, 15% of adolescents in the 10th grade reported that they had been victims of sexual abuse. This is in line with a previous study in Iceland [6] as well as a global meta-analysis [3]. The same applies to the difference in the report of sexual abuse of boys and girls, with girls reporting it twice as often as boys. Our results show that low family affluence increases the likelihood of adolescents to experience sexual abuse. In particular, girls living in low affluent families seem to be affected by sexual abuse. 

Although serious mental health issues may originate before the teenage years, symptoms increase substantially during this period. This increase in symptoms may be partly attributed to negative psychological experiences, such as sexual abuse, occurring more frequently in adolescence. Since adolescent girls are much more likely to experience the trauma of sexual abuse than boys, it is clear that they are also at more risk for a mental health disadvantage from an early age than boys [24]. 

Few studies have focused on family affluence and sexual abuse in adolescence. Some have found a higher risk of sexual abuse reported by the least affluent adolescents [10,11], whereas one study reported no significant difference in childhood sexual abuse rates between non-poor and poor families [15]. The difference in these findings could stem from differences in the research methodology, as Oshima et al.’s data [15] come from childhood sexual abuse reports to the child protective services. 

Low socioeconomic status is an indicator of social disadvantage; for females it may independently contribute to the risk of sexual abuse. The double jeopardy hypothesis proposes that two or more concurrent sources of social disadvantage may interact to produce particularly negative outcomes. The detrimental effects of SES may thus be more potent for adolescent girls than for boys [33,34]. Indeed, the results of the present study seem to support this line of thought.

The measurement of perceived family wealth in the current study is of course a more subjective estimate of the adolescents’ economic situation than the family affluence scale (FAS), which assesses absolute material wealth. However, the HBSC questionnaire includes both these approaches. Using the German HBSC data, Moor et al. [35] found that both measures could identify social inequalities in adolescent health. A comparative study between Belgium and the Czech Republic [36] showed that while adolescents’ life satisfaction was positively related to both family affluence (FAS) and perceived family wealth, the latter was a much stronger predictor. Similarly, a Swedish study by Ahlborg et al. [28] showed that the perception of family wealth was more strongly related to health outcomes than the measures of material possessions. Using a large Nordic representative sample, Torsheim et al. [16] also found that perceived socioeconomic status was a stable measurement for socioeconomic status in relation to self-rated health in adolescents. In a relatively wealthy country like Iceland there is a ceiling effect for the FAS scale, since most homes are large and well equipped. It is therefore more relevant to ask about perceived family wealth as an estimation of social standing than FAS. Children have a good sense of their families’ affluence relative to others and these evaluations are strongly related to their individual health ratings [16].

Gender difference in the sexual abuse of adolescents does not manifest solely in prevalence. Some studies have suggested that boys and girls deal with the consequences differently. Chandy et al. [37] reported gender difference in response to sexual abuse. Adolescent girls reporting a history of sexual abuse seem to engage in internalized behavior, such as disordered eating, suicidal ideation, and other erratic behavior. Boys, on other hand, seem to engage in externalized behavior such as poor school performance, sexual risk taking, and delinquent activities. Chandy et al. [37] also report findings on gender differences in protective factors enhancing resiliency in sexually abused adolescents. Female adolescents having higher than average emotional attachment to their families are better off health-wise than those feeling spiritual or religious, as well as those with both parents at home. For boys, the protective factors were higher maternal education and a strong sense of parental concern for them. 

Mohler-Kuo et al. [7] suggested that while the prevalence of the more severe forms of sexual abuse of youths has been stable over time, sexual abuse without physical contact has increased, e.g., via the Internet or various types of messaging. Moreover, they cite a dramatic rise of juvenile perpetrators of sexual abuse. 

Future research on family affluence and childhood sexual abuse needs to focus on protective factors for children, especially girls in less affluent families regarding the risk of being sexually abused during childhood. Ethnicity is also a relevant topic to pursue in this context. Furthermore, increased focus should be placed on the gender and age of the perpetrators. 

### Limitations

The present study does not include the adolescents’ report on when the abuse occurred, e.g., in earlier childhood or closer in time to the data collection. Previous studies show an increase in the prevalence of self-reported sexual abuse among older adolescents. Current data does not include the age when the abuse happened, or whether abuse occurred repeatedly. However, these variables, along with the type of abuse, are important in the study of sexual abuse in children and adolescents and could be important for deeper and further understanding of how and why the least affluent adolescents are at greater risk for sexual abuse than more affluent adolescents.

Studies based on self-reported data gathered in a school setting always run the risk of under-reporting the scope of problems. High-risk students are more likely to be absent than others. Similarly, not all students answer the questions, and, as we see from our previous study [5], those who do so are more likely to report more risk behavior and less well-being, raising the question whether their non-response is the result of a traumatic experience.

The current study did not consider the impact of neighborhood affluence. However, recent studies have shown this to be a relevant factor in the exposure of children and youth in various risk behavior and situations affecting their health and well-being [14].

## 5. Conclusions

Our results show that girls were more than twice as likely to be sexually abused as boys. It also demonstrated that adolescents perceiving their families to be less well off than others were twice as likely to report sexual abuse as those of ample or medium family affluence. But family affluence had much more effect on the prevalence of abuse of girls than of boys. These results stress the vulnerability of children, especially girls, living in less affluent homes to CSA. Given the seriousness of the impact CSA has on the future health and well-being of victims, they also highlight the urgency of supporting the families of these children economically. Politicians, as well as education, welfare, and health professionals have an important role in supporting less affluent children and their families. It is also important to identify and try to treat the perpetrators in the children’s environment who commit CSA. A significant number of them may belong to the same peer group. Results from the current study and others, will hopefully lead to more effective prevention and treatment of the parties involved.

## Figures and Tables

**Table 1:** Prevalence of sexual abuse in 15-year olds in Iceland (percentages and number).

Forms of Sexual Abuse	Boys	Girls
(a) Has someone touched or fondled you in a sexual way when you did not want them to?		
I refuse to answer		
Never	3.9 (64)	4.2 (67)
Once	89.8 (1460)	78.9 (1258)
A few times	2.4 (39)	9.4 (150)
Many times	1.9 (31)	2.1 (33)
(b) Has someone made you touch their body in a sexual way when you did not want them to?		
I refuse to answer	3.6 (59)	2.6 (41)
Never	92.4 (1501)	89.2 (1423)
Once	1.8 (30)	5.3 (84)
A few times	1.2 (19)	1.9 (31)
Many times	0.9 (15)	1.1 (17)
(c) Has someone attempted oral, anal, or vaginal intercourse with you when you did not want them to?		
I refuse to answer	3.0 (49)	3.3 (52)
Never	90.9 (1474)	84.5 (1345)
Once	2.7 (43)	7.2 (114)
A few times	2.0 (32)	3.8 (60)
Many times	1.5 (24)	1.3 (21)
(d) Has someone actually had oral, anal, or vaginal intercourse with you when you did not want them to?		
I refuse to answer	3.3 (54)	3.1 (50)
Never	92.7 (1504)	90.3 (1437)
Once	1.5 (24)	4.0 (63)
A few times	1.3 (21)	1.4 (22)
Many times	1.2 (19)	1.2 (19)

**Table 2:** Prevalence of sexual abuse by perceived family wealth and gender (percentages and number).

Perceived Family Wealth	Never Abused	Abused
Total		
Well off	87.0 (1870)	13.0 (280)
Average	84.2 (863)	15.8 (162)
Not well off	70.1 (138)	29.9 (59)
Boys		
Well off	90.3 (1047)	9.7 (112)
Average	92.5 (432)	7.5 (35)
Not well off	83.3 (60)	16.7 (12)
Girls		
Well off	83.0 (823)	17.0 (168)
Average	77.2 (431)	22.8 (127)
Not well off	62.4 (78)	37.6 (47)

**Table 3:** Binary logistic regression predicting the likelihood of sexual abuse in Icelandic adolescents.

Model	B	SE	OR	95% CI	*p*-Value
Model 1					
Constant	−2.138	0.097	0.118		<0.001
Family affluence	0.214	0.043	1.24	1.14–1.35	<0.001
Model 2					
Constant	−3.459	0.192	0.031		<0.001
Family affluence	0.179	0.044	1.20	1.10–1.31	<0.001
Gender	0.874	0.104	2.40	2.00–2.94	<0.001

B: intercepts, SE: standard error, OR: odds ratio, CI: confidence intervals.
